# **Denial and Distraction: How the Populist Radical Right Responds to COVID-19** Comment on "A Scoping Review of PRR Parties’ Influence on Welfare Policy and its Implication for Population Health in Europe"

**DOI:** 10.34172/ijhpm.2020.141

**Published:** 2020-08-03

**Authors:** Michelle Falkenbach, Scott L. Greer

**Affiliations:** ^1^Department of Health Management and Policy, School of Public Health, University of Michigan, Ann Arbor, MI, USA.; ^2^University of Michigan, Ann Arbor, MI, USA.

**Keywords:** Populist Radical Right, COVID-19 Pandemic, Public Health, Health Policy

## Abstract

This commentary considers the impact of the coronavirus disease 2019 (COVID-19) pandemic on the study of populist radical right (PRR) politicians and their influence on public health and health policy. A systematic review of recent research on the influence of PRR politicians on the health and welfare policies shows that health is not a policy arena that these politicians have much experience in. In office, their effects can be destructive, primarily because they subordinate health to their other goals. Brazil, the US and the UK all show this pattern. PRR politicians in opposition such as the Freedom Party of Austria (FPÖ) in Austria or the Lega in Italy, said very little during the actual health crisis, but once the public no longer appeared afraid they lost no time in reactivating anti-European Union (EU) sentiments. Whether in government or in opposition, PRR politicians opted for distraction and denial. Their effects ranged from making the pandemic worse.

Over the last decade, politicians of the Populist Radical Right (PRR) such as the Freedom Party of Austria (FPÖ) in Austria, Donald Trump in the United States, the Lega in Italy, Boris Johnson in the United Kingdom or Jair Bolsonaro in Brazil have established themselves as serious competitors in political and electoral systems worldwide. PRR politicians are defined as any party or single politician whose political style combines nativism (believing in an ethnically united people with a common territory), authoritarianism (believing in the value of obeying authority) and populism (preferring the ‘common sense’ of a unified people to ‘corrupt elite’ knowledge).^1,2^ Their presence has increased in the European Parliament and they have been in government on national and subnational levels across the globe.

Literature that addresses their actions in government, rather than just their words, tends to focus on their preferred issues: immigration, integration and security. It generally finds their policies to be consistent with their ideologies: nativist and authoritarian. PRR politicians in government lead to the adoption of strict anti-immigration policies, authoritarian integration programs and stringent legal reforms looking to benefit the native population over any outside group. But what about a policy such as health which does not fit well with the preferred rhetoric of PRR politicians? The impact of PRR politicians on health policies is largely neglected, despite the fact that some PRR politicians have been appointed Health Minister at the federal level^[1]^ while others have appointed a Health Minister at a regional level^[2]^. Even if PRR politicians do not want to talk about health, we can talk about what they do that affects health.

The 2020 published article entitled “A Scoping Review of PRR Parties’ Influence on Welfare Policy and its Implication for Population Health in Europe,” by Rinaldi and Bekker, focuses on exactly this point. The authors establish that there is little research “about the direct relationship between PRR parties and health.”^3^ In fact, they found the research surrounding health policies to be so thin that they had to expand their scope to include social policies. This combination of social and health policies led them to the conclusion that PRR parties impact welfare policies by implementing a welfare chauvinistic agenda that restricts access and eligibility to provisions for outsider groups such as immigrants and minorities.^3^

 With the coronavirus disease 2019 (COVID-19) pandemic, the world has been exposed to the biggest public health crisis to date, providing researchers with a new, and perhaps more direct chance to study PRR politicians and their impact on public health and health policy.

 In or out of power, the selected PRR responses were similar from country to country: denial and distraction. PRR politicians, particularly in the five countries discussed, sometimes went so far as to deny the existence of the pandemic, and insofar as they had a response it was to find someone to blame for the crisis, whether it be the European Union (EU), the World Health Organization (WHO) or migrants. This is consistent with a family of politicians who win few votes with healthcare and generally prefer to avoid health topics or reframe them as the kinds of security and immigration issues that the PRR prefers to emphasize.

## Populist Radical Right Politicians in Government: Jair Bolsonaro, Boris Johnson, and Donald Trump

At the end of January, Trump publicly dismissed risks of the coronavirus, stating that everything was under control. The White House did not exercise the leadership necessary to mobilize the federal government, notably by permitting an interagency conflict over test kits to paralyze testing; later, Trump would claim, and confirm, that “when you do testing to that extent, you’re going to find more people; you’re going to find more cases. So I said to my people, slow the testing down please.”^4^ Massive cuts were made to research and health institutions prior to the onset of the pandemic, so it is of no surprise that health professionals were lacking not only testing kits, but also reliable information and coordination efforts from the dispersed and underfunded agencies. The result: A public health disaster claiming the lives of well over 136000^5^ Americans to date (in the United States, like most countries, COVID-19 cases and attributable deaths are undercounts due to limited testing, so excess mortality is the useful statistic). Trump’s preferred reactions were focused on migration (eg, suspending visas) and border closures, nonsensical given that the virus was clearly endemic in the United States and people leaving the United States for other countries were a bigger global health threat than those entering. After protests erupted across the United States in favor of opening business, shops and restaurants again, Trump actively voiced his support for them, creating an even deeper divide between Democratic and Republican states. In addition, Trump blamed the WHO for their late reaction and announced that the United States would withdraw. By late June he had effectively abandoned the US response plan and dismantled the federal coordinating system, restarting rallies and changing the topic to his preferred issues of “law and order” (racist code in the United States) and a putative economic rebound.

 Similarly, UK Prime Minister Boris Johnson chose to ignore the gravity of the situation, despite having personally been hospitalized with it. Unforced but unsurprising English errors made the situation worse. Contact-tracing was stop-and-start, policy for schools in England was inconsistent and poorly explained, and communications were often unclear. Contracting testing to big outsourcing companies rather than running it through the National Health Service slowed the process and disconnected it from health services. Public health messaging was confusing (a government call to “stay alert” was never explained despite its being a key slogan) and undermined by the refusal to fire high-profile Johnson advisor, Dominic Cummings, who very publicly flouted quarantine. Having muddied and undermined its stay at home message, the Johnson government then courted the right-wing press and its voters by re-opening pubs at the end of June while also imposing a two week quarantine on international travelers, also nonsensical given that the United Kingdom was at that point an exporter rather than an importer of the virus.

Brazilian president Jair Bolsonaro was probably the most flagrant denialist. He is still, after registering almost half a million infections and being infected himself, denying the severity of the disease. Even after 17500 Brazilians were reported dead from the virus in mid-May, the President refused to take the matter seriously. He was quoted saying things like the virus was no more than a “little flu” and that China is responsible for provoking hysteria throughout the world.^5^ While the President continues to downplay the virus, regional authorities have taken charge and ordered lockdowns. Brazilian health ministers (there have been three within the course of a few months) have attempted to support these local measures of contagion, however, the President has taken to either firing them, forcing them to resign or chooses to blame them, and local authorities, for the stagnating economy. Brazil is second only to the United States with 1.23 million officially recorded corona infections, however the estimated number of unknown cases is speculated as being much higher.

 What these instances have proven is that the populist rhetoric, solidified in emotive narratives, is not the answer to a public health crisis. What becomes strikingly clear is that these three PRR politicians have very little competence in matters of public health and healthcare. Trump’s suggestion of injecting disinfectant to treat the virus and Johnson and Bolsonaro’s continued handshaking and close contact with people underlines their attempt to downplay the pandemic. Health is not a good issue for PRR politicians, and so it is unsurprising, if tragic, that they consistently try to change the topic rather than address the problem.

## Populist Radical Right Politicians in Opposition: Austria and Italy

 PRR politicians in opposition, such as the Austrian FPÖ or the Italian Lega, initially blamed migrants for its onset, pushed for early border closures and are now, as the number of infected continue to decrease, demanding a quick return to “normality” in order to save the economy. Freed from the responsibility of actually having to manage the pandemic, these politicians kept quiet while the virus infiltrated their respective countries. As soon as the health scare appeared to be over and public sentiment began questioning government measures, these same leaders reemerged unleashing their criticisms of governmnet measures as having been too harsh, undeomocratic and economically desastorous; therein casting blame in a way that increased the salience of their preferred issues.

Although not in government at the time, Lega head, Matteo Salvini, blamed Italy’s prime Minister Giuseppe Conte in February 2020 saying that he was not defending Italy and Italians from the coronavirus when a boat carrying African migrants was allowed to dock in Sicily. In fact, Salvini advocated for closing the borders entirely at that time. On April 30, 2020, several PRR Lega leaders including Salvini himself, occupied the Italian parliament in protest of ongoing lockdown measures demanding the “restoration of full liberties”^6^ despite the fact that the country was still reporting 1500 new infections per day.

Similarly, Norbert Hofer, leader of the PRR FPÖ in Austria called for border closures at the end of February, especially with Italy, contrary to WHO advice at the time as to how to contain the spread of the virus. 500 demonstrators joined the Viennese FPÖ in a protest against the “corona craziness” brought about by the ÖVP/Green government that massively “restricted civil liberties” through their “excessive corona measures.”^7^

Not only are the PRR leaders critizcing their country’s own government, but they are reigniting anti-EU sentiments. Salvini is advocating for re-founding the EU based on new principles so that each country can have its own monetary policy^8^ and the FPÖ is unsurprisingly against the EU proposed corona bonds.^9^

## Denial and Distraction

As Rinaldi and Bekker and the scholarship they review show, health is not a favored issue for the PRR. Security and migration are their preferred issues. Before the pandemic, the result was that they de-emphasized the topic, framed it in nativist terms when they did discuss it, and when they had to make health policies tended to pursue fairly conventional right-wing approaches.^10,11^

 Faced with a pandemic, their key strategies, whether in or out of power, were denial and distraction. Denial could be explicit, as with Trump’s claim that coronavirus was a “hoax,” or implicit, as in efforts to reopen countries before containing the outbreak. Distraction meant blaming somebody else, be it the EU, WHO, or foreigners, and led to damaging border control policies as well as the US decision to leave the WHO mid-pandemic. Both strategies undermine public health and cost lives. Both strategies reflect the PRR preference for nativist and authoritarian policies and issue framings that support those policies. PRR discomfort with health policy has significantly worsened the public health crisis and in some countries contributed to a crisis of democracy.

## Ethical issues

 Not applicable.

## Competing interests

 Authors declare that they have no competing interests.

## Authors’ contributions

 Both MF and SLG contributed to the conception, drafting, and review of this commentary.

## Authors’ affiliations

^1^Department of Health Management and Policy, School of Public Health, University of Michigan, Ann Arbor, MI, USA. ^2^University of Michigan, Ann Arbor, MI, USA.

## Endnotes

 [1] This was the case in Austria in 2000 and again in 2018. [2] Luca Coletto in Veneto, Italy has been the Minister of Health for the region since 2010.
